# Characterization of black pigment used in 30 BC fresco wall paint using instrumental methods and chemometry

**DOI:** 10.1186/1752-153X-6-S2-S2

**Published:** 2012-05-02

**Authors:** Tania Gatta, Luigi Campanella, Carlo Coluzza, Vania Mambro, Paolo Postorino, Mauro Tomassetti, Giovanni Visco

**Affiliations:** 1 Chemistry Department, University La Sapienza, P.le A. Moro, 5, 00185, Rome, Italy; 2 Physic Department, University La Sapienza, P.le A. Moro, 5, 00185, Rome, Italy

## Abstract

**Background and methods:**

Several standard powdered black pigments were characterized by means of thermogravimetry TG-DTG and allied techniques. These pigments were used to make standard plaster frescoes at this purpose prepared. The latter ones were subjected to Raman and reflectance analysis. The results obtained, together with TG data, were chemometrically processed and used to identify an analogous standard fresco fabricated by an unknown commercial black pigment, obtaining excellent results.

**Results:**

The same colorimetric and reflectometric techniques, coupled with suitable chemometric techniques, were then successfully used to identify the type of black pigment present in an ancient roman fresco of the Imperial Age (30 B.C.).

**Conclusion:**

TG-DTG resulted useful techniques to autenticate powdered black pigments.Colorimetry and Raman, but also the only colorimetry, were useful to identify an ancient black pigment in situ.

## Aim

The aim of the present research was to identify the black pigment used in a roman fresco dated to 30 B.C. using a non destructive method.

## Background

The application required comparison with pre-recorded reflectance spectra of standard plaster frescoes ad hoc prepared.

For the preparation of these “standard frescoes” the previous characterization of utilized different standard black pigments was necessary and was performed by means of different instrumental techniques, particularly, thermogravimetry and differential thermoanalysis (TG-DTG).

On the other hand, in order to characterize and identify a standard fresco prepared using an unknown black pigment, thermogravimetry, Raman microspectroscopy and colorimetry were applied, while to identify the in situ Roman fresco, it was possible to apply only colorimetry and reflectance spectroscopy.

Lastly, Multivariate Analysis techniques were used in order to compare all the available instrumental data.

## Results and discussion

**Figure 1:**
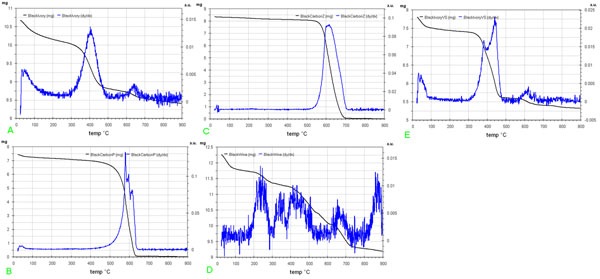
TG and DTG curves of powdered standard black pigments purchased in specialized shops: (a) black-ivory; (b) black-carbon (P); (c) black-carbon (Z); (d) black-wine; (e) black-Ivory- (VS) considered unknown black pigment sample.

Thermogravimetry seems to be the most useful technique to characterize the purchased powdered standard black pigments. The TG and DTG curves values obtained are shown in Fig.1, while the TG data and relative activation energy values (E_a_) are summarized in Table 1.

**Table 1:** Thermogravimetric data and activation energy values (E_a_) relative to single steps (all values are the mean of three determinations; for the temperature values RSD%≤0.5%; for the mass loss values RSD% ≤0.1%; for the E_a_ values RSD% ≤ 0.2%).

Samples	Loss of H_2_O		Step 1									***Step 2***					Res. % at 900 °C
		
			(sub step1a)						(sub step 1b)			(sub step2)					
	T [°C]	Mass Loss%	T [°C]	Mass Loss%	Ea [KJ/mol]	T [°C]	Mass Loss%	Ea [KJ/mol]	T [°C]	Mass Loss%	Ea [KJ/mol]	T [°C]	Mass Loss%	Ea [KJ / mol]	T [°C]	Mass Loss%	T[°C]

**Black-Ivory**	28					251						560					
	46	4.9				404	11.6					654	1.8				78.8
	250					590						695					
				
						250		113.2				560		216.4			
						540						720					
																	

**Black-Carbon P**	23					340											
	40					580	97.0										0.0
	50	2.4				638											
		
																	
						365		247.8									
						606											

**Black-Carbon Z**	23					500											
	30	0.01				615	99.0										0.0
	50					705											
		
																	
						512		247.5									
						615											

**Black-Vine**	23		170			375			480			625			770		
	60	4.3	260	3.6		440	6.6		490	2.8		700	5.6			2.0	74.9
	170		375			490			600			770			900		
		
																	
			170		105.1	500		375	550		172.0	625		212.4			
			375			550			625			770					

**Black-Ivory VS**	27					250						555					
	45	3.5				400	14.3					560	4.6				74.9
	245					502						690					
				
																	
						250		107.1				555		211.2			
						500						710					

More in detail:

(i) Thermogram of Black-Ivory: step at T<150 °C is due to water loss; step at T~400 °C is due to the oxidation of the carbonaceous material, deriving from the proteic material combustion; step at T≈640°C is related to the decomposition of calcium carbonate CaCO_3_→CO_2_↑+CaO, included in the hydroxiapatite, that is carbonated hydroxiapatite [1].

(ii) Thermograms of two different Black Carbon samples: very small step at T<100 °C is due to the water loss; step at T≈600 °C is the only important process due to the oxidation of Carbon to CO_2_, but DTG curves of two samples are different, probably due to the different granulometry of carbon particles. The sample from Z supplier seems more homogeneous, in fact the DTG peak of the oxidation process, at around 600 °C, is practically symmetric and more regular than the corresponding peak of sample from supplier P. As a conclusion sample Z has probably a granulometry more homogeneous than sample P. Lastly after the step at 900°C no residue is observed in the both cases.

(iii) Thermogram of black vine: step at T≈50 °C shows a fair amount loss of water; step at temperature between 250 and 370 °C can be attributed to the oxidation of carbonaceous and organic material, no totally burned, probably coming from cellulosic and ligninic material; step at temperature ranging from 600 to 750 °C is due to the decomposition of CaCO_3_. Finally, the steps at around 450 °C and 490 °C are respectively due, to the presence of hydrated silicates included in black vine pigment during the combustion process of vine wood sample, probably contaminated by silica dust and to the carbon oxidation.

(iv) Finally the steps and the behaviours of TG and DTG curves of the unknown considered sample (i.e. the black-ivory-VS) are very similar to those ones of black ivory standard samples. Looking at the Tab.1 we can also observe that activation energy (E_a_) data are more useful than temperature and mass loss data to study the authenticity of a powdered black pigment. Really temperature data are strongly influenced by granulometry and by the mass value of the sample. On the contrary E_a_ results constant when referred to the same process also even if this process occurs in different samples. At this purpose we can observe the case of the oxidation step in two different black-carbon samples, for which E_a_ is always ≈ 247 kJ/mol, also when the temperatures of two steps are relatively different. Similar observations can be performed for E_a_ values of oxidation process of carbon material in black-ivory and in black-vine. Lastly this consideration appears still very true for values of E_a_ at the decomposition process of small quantities of calcium carbonates included in the two alone pigments (about 210 kJ/mol) in both cases.

**Figure 2:**
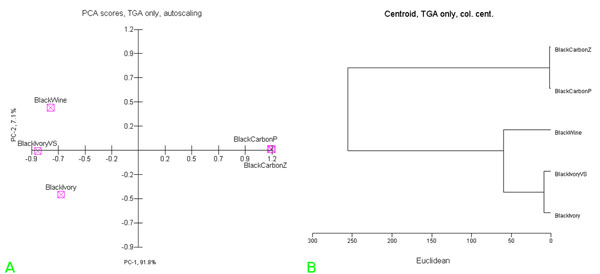
(a): PCA representation of scores of TG-DTG data of all powdered standard black pigments and of the black-ivory-(VS) considered as unknown pigment sample. (b): HCA representation of TG-DTG data of powdered standard black pigments and the considered unknown black pigment sample (i.e. black-ivory-(VS)).

Lastly, Multivariate Analysis techniques, i.e. Principal Component Analysis (PCA) and Hierarchic Cluster Analysis (HCA), were used in order to compare all the available thermogravimetric data [2]. The PCA and HCA representation of these TG data after column centering (see data set in “additional file 1, Tab. A”), are shown in the figures 2(a) and 2(b) respectively. It is interesting that the classification as black-ivory, for the unknown black sample before considered only as “probably”, was strongly corroborated [3,4].

EDS and FT-IR data in some cases confirmed the thermogravimetric results (see tables 2 and 3); for instance the presence of calcium carbonate in black-ivory and calcium and magnesium carbonate in black-vine; hydroxiapatite in black-ivory and iron oxides and silica traces in black-vine.

**Table 2:** EDS (Energy Dispersion Spectroscopy) data of standard black-ivory and black-vine samples.

Black-Ivory	C ++++ P + O ++ Si n Ca + + +
Black-Vine	C ++++ Fe ++ O ++ Ca + Si +++ S n Mg ++ Mn n

Legend of concentrations:

++++ = very high

+++ = high

++ = medium

+ =low

n = negligible

**Table 3:** IR (Infra-Red) absorption data of standard black-ivory and black-vine samples.

Black-Ivory [cm^-1^]	Attribution	Black Vine [cm^-1^]
	M-O (FeO)	420
	M-O (FeO)	451
	M-O (FeO)	462
550	PO_4_^3-^	
582	PO_4_^3-^	
625	CO_3_^2-^	626
724	CO_3_^2-^	725
873	CO_3_^2-^	
1018	CO_3_^2-^	1017
1107	CO_3_^2-^	

Before identifying the black pigments used in the ancient fresco, it was necessary to know if analytical methods and chemometric techniques used in the present research were capable of effectively distinguishing some top-quality purchased black pigments by which the standard frescoes were prepared. To check the validity of the analytical and chemometric procedures used, all the purchased standard pigments and the standard frescoes prepared by these pigments including the black-ivory-VS pigment, supplied as only “probable” and practically still considered by us as an unknown sample, was tested using three analytical techniques: Thermogravimetry, Colorimetry (Table 4) and Raman microspectroscopy (Table 5) [5].

**Table 4:** Colorimetric (CIELab) data of all standard frescoes fabricated using standard black pigments and the considered unknown black pigment (i.e. black-ivory-(VS)).

Identification of CIELab parameters of standard black pigments in a standard frescoes				
*Sample*	*Illuminating*	***L***	a*	b*

Black-Ivory	D 65 D 50	29.71±1.34 27.44±2.57	0.11±0.02 0.08±0.03	-0.29±0.09 -0.37±0.16

Black-Carbon P	D 65 D 50	33.12±1.11 32.87±0.54	-0.29±0.03 -0.76±0.03	-3-15±0.24 -3.33±0.21

Black-Carbon Z	D 65 D 50	41.28±0.65 40.96±0.61	-0.44±0.02 -1.05±0.04	-4.15±0.09 -4.24±0.15

Black-Vine	D 65 D 50	29.15±0.61 29.37±0.46	0.16±0.02 0.21±0.03	0.38±0.14 0.35±0.12

Black-Ivory VS	D 65 D 50	29.41±0.34 27.81±0.46	0.21±0.04 0.23±0.03	0.35±0.14 0.44±0.12

Measurements performed by spectrophotometer Minolta CM2600d, standard observatory 2°, MAV mask. Illuminants: D 65 (European standard) and D 50 (American standard).				

**Table 5:** Raman microspectroscopy data of standard frescoes fabricated using standard black pigments and the considered unknown black pigment (i.e. black-ivory-(VS)).

Identification of characteristic Raman peaks		
	[cm^-1^]	[cm^-1^]
**Black-Ivory**	*Experimental* *1346 m* *1594 s*	*Bibliographic reference* (*1*) *1354 s* *1609 s*

**Black-Carbon P**	*Experimental* *1337 s* *1587 s*	*Bibliographic reference* (*1*) *1320 s* *1595 s*

**Black-Carbon Z**	*Experimental* *1338 s* *1590 s*	*Bibliographic reference* (*1*) *1320 s* *1595 s*

**Black-Vine**	*Experimental* *-* *1580 s*	*Bibliographic reference* (*1*) *1350 w* *1573 s*

**Black-Ivory VS**	*Experimental* *1349 s* *1588 s*	*Bibliographic reference* (*1*) *-* *-*

LEGEND: *s* = strong signal, *m* = medium signal, *w* = weak signal

(1)Values reported by University of Florence. Relative acquisitions were obtained with laser line x=514.5 nm, lens 100x, time of acquisition 100”, resolution 7 cm^-1^.

We effected our measures with an instrument Lab Ram Infinity by Jobin Yvon Horiba Group, laser He-Ne (λ=632.8 nm), filter D06, hole100, grid 1800 strips/mm, resolution 3 cm^-1^, acquisition 5x60”

**Figure 3:**
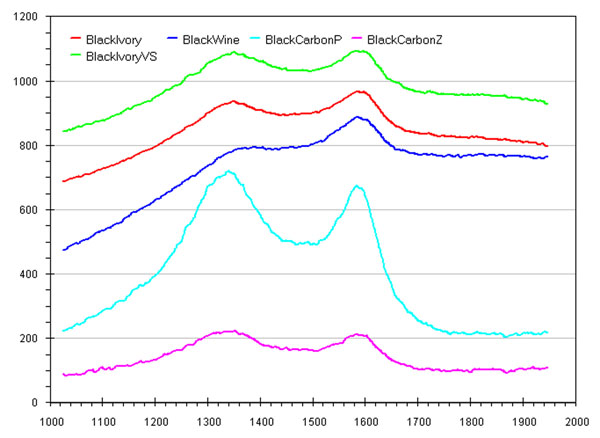
Raman spectroscopy curves of standard frescoes fabricated using standard black pigments and the considered unknown black pigment sample (i.e. black-ivory-(VS)).

It can be observed as the Raman microspectroscopy technique proves to be a good tool for recording the presence of any black carbon pigment in frescoes. However the wavelength differences of Raman peaks of different black pigments we studied were found to be in practice not always well obvious (Figure 3) [6].

**Figure 4:**
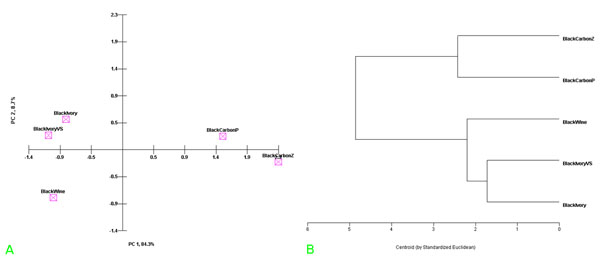
(a): PCA representation of the scores of data set values reported in Tab. 6, i.e. CIELab, TG-DTG and Raman data of all the standard black pigment frescoes fabricated in our laboratory and of the considered unknown black pigment sample (i.e. black-ivory-(VS)). (b): HCA representation of the data set values reported in Tab. 6, i.e. CIELab, TG-DTG and Raman data of all the standard black pigment frescoes fabricated in our laboratory and the considered unknown black pigment sample (i.e. black-ivory-(VS)).

Briefly, colorimetric, Raman and thermogravimetric data, referring respectively to standard frescoes, including that one of black pigment considered uncknown, were digitized using modern smoothing techniques (see data set in “additional file 1, Tab. B”) and processed by chemometric software (see PCA and HCA representation in Figs.4(a) and 4(b)). It was thus possible to identify definitively the unknown black pigment, which can be considered certainly as another black-ivory sample (confidence level 80 %).

**Table 6:** Comparison of colorimetric CIELab data of standard fabricated frescoes using standard black pigments and the same data found for the black pigment of the old Roman fresco.

Identification of a black pigment used in the old Roman fresco by means of CIELab parameters (values are the mean of three determinations)				
*Sample*	*Illuminating*	***L***	a*	b*

Black-Ivory	D 65 D 50	29.71±1.34 27.44±2.57	0.11±0.02 0.08±0.03	-0.29±0.09 -0.37±0.16

Black-Carbon P	D 65 D 50	33.12±1.11 32.87±0.54	-0.29±0.03 -0.76±0.03	-3-15±0.24 -3.33±0.21

Black-Carbon Z	D 65 D 50	41.28±0.65 40.96±0.61	-0.44±0.02 -1.05±0.04	-4.15±0.09 -4.24±0.15

Black-Vine	D 65 D 50	29.15±0.61 29.37±0.46	0.16±0.02 0.21±0.03	0.38±0.14 0.35±0.12

Black old Roman fresco	D 65 D 50	28.81±0.89 28.54±0.34	0.42±0.05 0.52±0.04	2.28±0.17 2.88±0.19

Measurements performed by spectrophotometer Minolta CM2600d, standard observatory 2°, MAV mask. Illuminants: D 65 (European standard) and D 50 (American standard).				

Lastly to characterize and identify old Roman black pigment (30 B.C.) only colorimetric data (that is the L*, a*, b* CIELab parameters), obtained by measurement in situ using Minolta software was possible to compare with those ones obtained by similar colorimetric measurements on standard frescoes, using chemometric methods (see Table 6).

**Figure 5:**
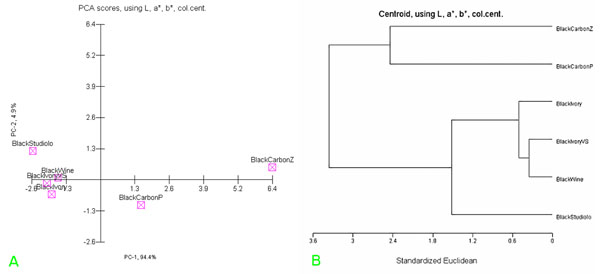
(a): PCA representation of the CIELab colorimetric data of all standard frescoes fabricated using standard black pigments and unknown black pigment of old roman fresco (i.e. black-studiolo). (b): HCA representation of CIELab colorimetric data of all standard frescoes fabricated using standard black pigments and unknown black pigment of old roman fresco (i.e. black-studiolo).

CIELab data of standard frescoes and those ones of old roman fresco were elaborated using PCA and HCA analysis (see data set in “additional file 1, Tab.C”). In practice using the chemometric representation of colorimetric data, displayed in Figs. 5(a) and 5(b), we were able to corroborate that the black pigment of the old Roman fresco under test was probably a black vine pigment. However, in this case it was not possible to include in the data matrix also Raman [7] and thermal analysis data of old roman black pigment, so that the identification in this case had a confidence level of not more than 65 %.

**Figure 6:**
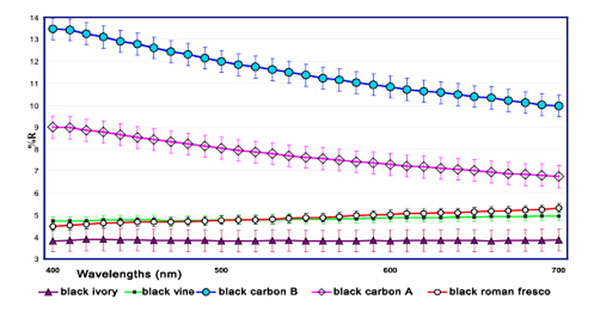
Reflectance spectra, in the visible field (400-700 nm) of all the standard frescoes fabricated in our laboratory using standard black pigments and reflectance spectrum of the black pigment of old Roman fresco recorded in the Augustus small study room.

On the other hand the Minolta instrument, being portable, could also allow to record in situ the reflectance spectra in the visible region (400-700 nm), therefore it was possible to compare the reflectance spectrum of the black pigment of the old Roman fresco with the spectra of all well characterized standard black pigments in the standard frescoes (see figure 6 in which it is possible to stress as the reflectance spectrum of the black-vine pigment of the standard fresco almost completely is overlapping that one of the black pigment recorded in the Augustus’ small study room). Indeed, the very small differences between the spectra of ancient and modern pigments are justified by the presence of wax (normally used in Roman frescoes to protect from moisture and to increase colour brightness) and by aging processes [8].

## Conclusions

In conclusion, thermogravimetry seems to be the most useful technique to characterize and authenticate powdered black pigments, while, by using only the visible reflectance trend and colorimetric parameters, processed by chemometry, we were still able to identify an ancient pigment in situ. Of course in the lucky case in which all the three techniques (colorimetry, Raman and thermogravimetry) can be simultaneously applied, the identification is more simple and sure. The limitation of the present approach is due to the necessity of record standard frescoes spectra as reference.

## Experimental

### Samples

**Figure 7:**
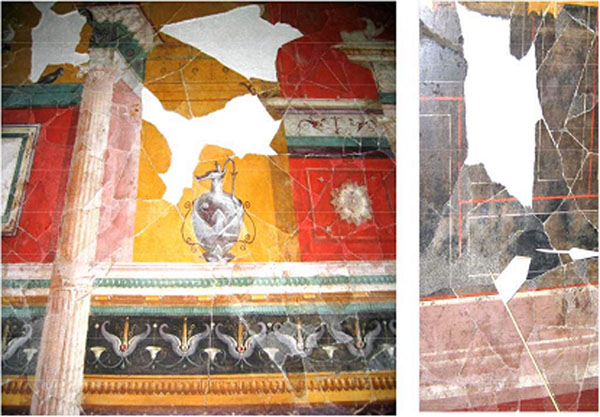
Photo of particulars in the Augustus small study room (Palatino, Rome – 30 B.C.)

The fresco we studied was found on the Palatine in Rome after the excavation of “Emperor Octavianus’ house”, in the room called “Augustus’ small study room (Fig.7)”. In the present work the research was restricted to black pigment.

**Figure 8:**
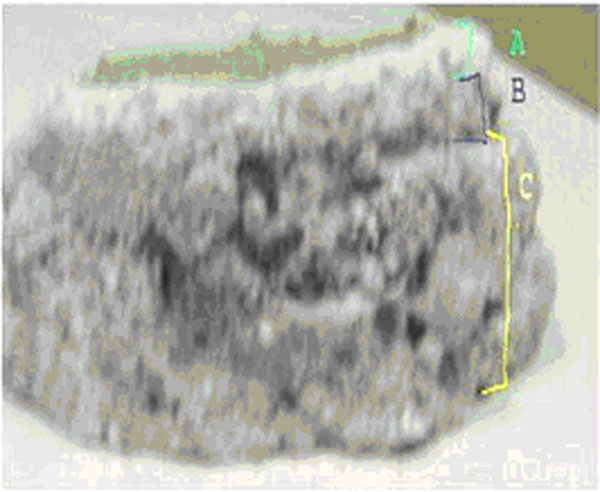
Typical standard frescoes fabricated in our laboratory

**Figure 9:**
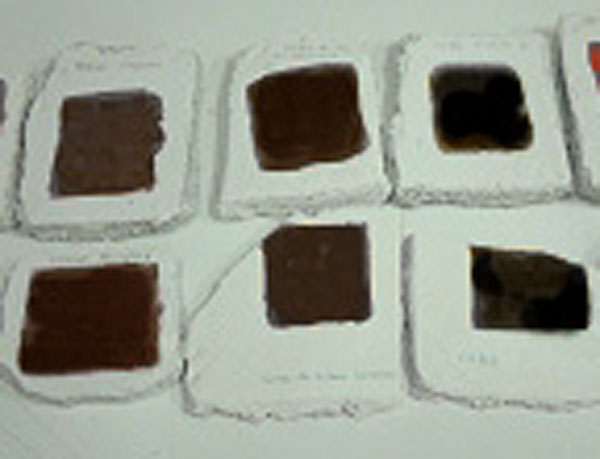
“Tectorium” of the standard frescoes fabricated in our laboratory

It is well know as in roman frescoes three different types of black pigments were especially used: black-ivory, black-carbon and black-vine [9,10]. At our recognizing purpose we bought all these pigments. To recognize the old roman black pigment we took colorimetric and reflectance spectrophotometric measures on standard frescoes which we prepared in laboratory (Fig.8) using standard pigments, according the peculiar Roman technique reported in literature [9]. To obtain the final plaster (“tectorio”) to be painted we fabricated several layers of different composition (Fig.9). Our samples consisted of three layers: the first one (arriccio) of pozzolana, lake sand, and slaked lime, the second one (intonaco), opportunely spluttered, consisted of lake sand (thinner) and slaked lime, the last one (intonachino) of few millimeters size, of slaked lime and marble's powder; it was polished to improve the reflectivity [11].

Standard black pigments were purchased over the counter in Rome and Florence in shops specializing in the sale of restoration materials. The standard black pigments available on the market and used to construct the training set in the chemometric process were: one black-ivory, two black-carbon and one black-vine pigments. To reach our aim we used these samples because they are the only certified samples available on the Italian market.

Lastly, another black pigment, supplied to us (but “only probably”) as black-ivory, was considered as an unknown sample and used for the “Validation Set”, therefore name black-ivory-VS.

## Thermogravimetry

We used thermogravimetry (TG, DTG) to characterize standard powdered pigments. The thermogravimetric (TG) curves show the variations of the percent mass of the sample as a function of increased temperature, therefore the heating generally can determine variations (losses) of the sample mass. The temperatures of these processes are typical for each sample [12]. The DTG curves show the first derivative of the TG curves. These measures were carried out by a thermobalance Mettler TG 10-TA in the following conditions: dynamic air, flow rate 10 ml/min, heating rate 10°C/min from 25 to 900°C, the sensitivity of the balance was 1 μg. It was experimentally determined also the activation energy (E_a_) of the main TG steps. E_a_ was calculated from a single dynamic thermogravimetric measurement using a multiple linear regression (n^th^ order kinetics) method (i.e. the Wyden-Widmann’s method [13]), by means of the following Arrhenius type equation: dα/dt = k_0_ e-E_a_/RT (1-α)n. In the applied least square method the sum of the squares of the differences between the dα/dt values obtained from the above mentioned equation and values derived from the TG and DTG measurements, respectively taking into account that: α = ∆m/∆m_tot_ and dα/dt =(dm/dt)/∆m_tot_, attains its minimum values for the selected values of k_0_, n and E_a_.

## EDS and FT-IR

Using an energy dispersion spectrometer (EDS) as detector we obtained the chemical analysis of standard pigments by scanning electron microscopy, measuring the energy and distribution of the intensity of X photon generated by the electronic beam of the (SEM) electron microscopy, of which the sample under analysis is the target. Atoms having atomic number lower then that one of sodium are not revealed. The IR spectra were obtained by a spectrophotometer FT-IR Perkin-Elmer NIR. At this purpose 2-3 mg of each pigment were dispersed in 100 mg of anhydrous KBr, then obtaining tablets to be analyzed.

## Raman microspectroscopy

For Raman microspectroscopy used to characterize our standard frescoes a Lab Ram Infinity (Jobin 1024x256 pixels, Yvon Horiba Group) was used with 25 mW of maximum CCD (Charge Couplet Device) light stimulation, origin of an electric signal. The laser attenuation is given: 1-2% by mirrors, 1-2% by lenses, 50% by beam splitter, 5% by the lens and for a 10^-6^ factor by the notch filter. The laser beam power may be reduced by the regulation of opportune filters which are in order: (starting from the filter with maximum attenuation) D3-D2-D1-D06-D03-without filter (according the signal attenuation factor due to the selected filter, for example the D03 filter reduces the signal power of a factor 10^3^). The high intensity of the laser beam produces a local overheating which can damage the sample modifying its structure. In the case of black pigments (essentially combustive materials) the risk is minimum and it is sufficient to use a filter D03. For the instrument calibration neon was used. For black pigments the only interesting region is the one included in the frequency range of 1200 – 1600 cm^-1^ (8333.3-6250 nm). We utilized a grid with 1800 strips/mm pointed on the frequency of 1500 cm^-1^ (6666.7 nm), D03 filter, 50x lens, hole 100. The definitive spectrum was the average of five measures, each one being recorded in 60”.

## Visible reflectance spectroscopy and colorimetry

To identify and characterize the black pigments of frescoes we used the colorimetric and reflectometric techniques. At this end colorimetric spectrophotometer Minolta CM2600d allowed us to perform reflectance measurements in the visible region (400-700 nm). It was so possible to determine both the reflectance curve and the values of colorimetric parameters. We measured each commercial pigment on 12 different zones on the standard fresco, obtaining 12 reflectance spectra, of which we calculated the average and the relative standard deviation. It is well known that there are many different ways to define each colour; the more used is the CIELab [14], by which each is defined by means of three parameters: L*, a*, and b*. These three parameters are in mathematical connection with Munsel parameter defined as L* (the luminance), H*=arctang (b*/a*) (the hue), and C*=(a*2+b*2)1/2 (the saturation or chromaticy). In order to define a true colour we used an "observer of 2° ", that corresponds to cones vision, defined by CIE (Commission Internationale de l'Enclairage) since 1931.

## Chemometric methods

For chemometrics the software used was: Lotus spreadsheet 9.8 (IBM/Lotus, Usa); Past 2.14 (free version by Øyvind Hammer, Norvay); Datalab 2.99 (light version by H. Lohninger, Austria); Multivariate Analysis (an Excel ad-in by Prof. R. G. Brereton).

## Competing interests

The authors declare that they have no competing interests.

## Authors’ contributions

TG collected data and helped draft the manuscript, LC coordinated the research group, CC dealt with colorimetric data, VM performed colorimetric and Raman measurements and fabricated standard frescoes, PP dealt with Raman data, MT performed thermogravimetric measurements and wrote the paper and GV dealt with chemometric processing of data.

## Supplementary Material

Additional file 1 Table A: Table_A., *.doc, data set of TG-DTG data after col. cent , “data set of TG-DTG main data of standard powdered black pigments, used to corroborate the identification of black-ivory -VS pigment considered as unknown pigment.” Table B: Table_B, *.doc, data set of CIELab, TG-DTG and Raman data, “data set of CIELab, TG-DTG and Raman data, used to identify (after column centering) the “black-ivory-VS” considered an unknown sample.” Table C: Table_C, *.doc, data set of colorimetric CIELab data, “data set of colorimetric CIELab data used for the identification of the type of black pigment of old Roman fresco.” Click here for file
