# Supplementary material for: Characterization of black pigment used in 30 BC fresco wall paint using instrumental methods and chemometry
Source: Chem Cent J. 2012 May 2;6(Suppl 2):S2. doi: 10.1186/1752-153X-6-S2-S2 (PMC3395117; doi:10.1186/1752-153X-6-S2-S2)
Supplement: Additional file 1 — Table A: Table_A., *.doc, data set of TG-DTG data after col. cent , “data set of TG-DTG main data of standard powdered black pigments, used to corroborate the identification of black-ivory -VS pigment considered as unknown pigment.” Table B: Table_B, *.doc, data set of CIELab, TG-DTG and Raman data, “data set of CIELab, TG-DTG and Raman data, used to identify (after column centering) the “black-ivory-VS” considered an unknown sample.” Table C: Table_C, *.doc, data set of colorimetric CIELab data, “data set of colorimetric CIELab data used for the identification of the type of black pigment of old Roman fresco.” [file 1752-153X-6-S2-S2-S1.doc]

|  | **Step 1** | | **Step 2** | | **Res% at 900°C** |
| --- | --- | --- | --- | --- | --- |
| **Black-Ivory** | **loss%** | **KJ/mol** | **loss%** | **KJ/mol** |  |
| **Black Carbon P** | 16.5 | 113.2 | 18.3 | 216.4 | 78.8 |
| **Black Carbon Z** | 2.4 | 30 | 99.4 | 247.8 | 0.1 |
| **Black Wine** | 0.1 | 20 | 99 | 247.5 | 0.1 |
| **Black-Ivory VS** | 17.3 | 172 | 22.9 | 212.4 | 74.9 |
| **Black-Ivory** | 24.3 | 215.1 | 29.8 | 221.2 | 68.2 |

Table A

Data set after col.cent.

|  | **CIEL ab data** | | | | | | **Thermogravimetric data** | | | | **Raman data** | | |
| --- | --- | --- | --- | --- | --- | --- | --- | --- | --- | --- | --- | --- | --- |
|  | **D65 L** | **D65 a*** | **D65 b*** | **D50 L** | **D50 a*** | **D50 b*** | **loss1%** | **KJ/mol** | **loss2%** | **KJ/mol** | **Res%** | **Rpeek1** | **Rpeek2** |
| **Black Ivory** | -2.203 | 0.082 | 0.473 | -3.725 | 0.208 | 0.342 | 4.380 | 3.140 | -35.580 | -12.660 | 34.380 | -0.800 | 4.600 |
| **Black Carbon P** | 1.207 | -0.318 | -2.387 | 1.705 | -0.632 | -2.618 | -9.720 | -80.060 | 45.520 | 18.740 | -44.320 | -9.800 | -1.400 |
| **Black Carbon Z** | 9.367 | -0.468 | -3.387 | 9.795 | -0.922 | -3.528 | -12.020 | -90.060 | 45.120 | 18.440 | -44.320 | -10.800 | -3.400 |
| **Black Wine** | -2.763 | 0.132 | 1.143 | -1.795 | 0.338 | 1.062 | 5.180 | 61.940 | -30.980 | -16.660 | 30.480 | 21.200 | -2.400 |
| **Black Ivory VS** | -2.503 | 0.182 | 1.113 | -3.355 | 0.358 | 1.152 | 12.180 | 105.040 | -24.080 | -7.860 | 23.780 | 0.200 | 2.600 |
| **Black Studiolo** | -3.103 | 0.392 | 3.043 | -2.625 | 0.648 | 3.592 |  |  |  |  |  |  |  |

Table B

|  | **Illuminating European Standard** | | | **Illuminating American Standard** | | |
| --- | --- | --- | --- | --- | --- | --- |
|  | **D65 L** | **D65 a*** | **D65 b*** | **D50 L** | **D50 a*** | **D50 b*** |
| **Black-Ivory** | -2.203 | 0.082 | 0.473 | -3.725 | 0.208 | 0.342 |
| **Black Carbon P** | 1.207 | -0.318 | -2.387 | 1.705 | -0.632 | -2.618 |
| **Black Carbon Z** | 9.367 | -0.468 | -3.387 | 9.795 | -0.922 | -3.528 |
| **Black Wine** | -2.763 | 0.132 | 1.143 | -1.795 | 0.338 | 1.062 |
| **Black-Ivory VS** | -2.503 | 0.182 | 1.113 | -3.355 | 0.358 | 1.152 |
| **Black Studiolo** | -3.103 | 0.392 | 3.043 | -2.625 | 0.648 | 3.592 |

Table C
